# Quantitative lipidomics reveals Lipid perturbation of plasma apoptotic extracellular vesicles during physiological aging

**DOI:** 10.3389/fcell.2026.1853081

**Published:** 2026-09-30

**Authors:** Maojiao Li, Haisen Huang, Qi Tang

**Affiliations:** 1 West China School of Public Health and West China Fourth Hospital, Sichuan University, Chengdu, China; 2 State Key Laboratory of Oral Diseases and National Clinical Research Center for Oral Diseases and Engineering Research Center of Oral Translational Medicine, Ministry of Education and National Engineering Laboratory for Oral Regenerative Medicine, West China Hospital of Stomatology, Sichuan University, Chengdu, China; 3 Novital Biotechnology Company, Limited, Chengdu, China

**Keywords:** aging, ApoEVs, lipid pathway, lipidomics, plasma

## Abstract

**Introduction:**

Aging is closely implicated in the pathogenesis of chronic diseases, degenerative conditions, and mortality, making it a core research focus in geroscience. As direct secretory products of apoptotic cells, apoptotic bodies can directly reflect the intensity of apoptotic activity and the degree of systemic and cellular aging. Given their close correlation with senescence progression, apoptotic bodies hold great potential as reliable and sensitive biomarkers for evaluating aging status. However, apoptotic bodies as effective indicators of aging remain to be explored.

**Methods:**

We characterized the lipid profile of apoptotic bodies (ApoEVs) and explored the changes in lipid species related to aging. Total lipids, multiple lipid subclasses, and fatty acid saturation within plasma apoptotic bodies were analyzed to identify aging-associated lipid alterations.

**Results and Discussion:**

A comprehensive reduction in total lipids, multiple lipid subclasses, and fatty acid saturation within plasma apoptotic bodies highlights systemic lipid reprogramming in aging. Decreased total levels of Hex2Cer, LPC, LPE, TG, ChE, SM, and SPH are the features of aging. This study not only deepens the understanding of aging-related lipid dysfunction, but also offers circulating lipid biomarkers for assessing biological aging and age-related pathologies.

## 1 Introduction

With the shifting demographics toward an aging population, a spectrum of debilitating age-related disorders has emerged, accompanied by escalating healthcare costs and mounting societal burdens. Advances in technological and biological research have broadened our understanding of aging and its associated pathologies. Heterochronic parabiosis—the surgical joining of young and old animals—has been shown to rejuvenate multiple tissues in the aged partner (Conboy et al., 2005; Liu et al., 2017; Conboy et al., 2013). This rejuvenation effect is thought to result from the exchange of biological cargoes via plasma, underscoring the critical importance of plasma in aging research. Plasma biomarkers offer a time- and cost-efficient means of detection, with the added benefits of being minimally invasive and readily accessible. Consequently, there has been a marked surge in clinical studies concentrating on plasma biomarkers linked to aging and age-related diseases, Accumulating studies have established a tight functional linkage between ApoEVs and cellular senescence. Defective apoptosis and reduced secretion of ApoEVs lead to accumulated DNA damage and premature senescence. ApoEVs carry molecular signatures reflecting the physiological state of parental cells, and their compositional remodeling faithfully mirrors systemic aging progression (Huang et al., 2024; Fu et al., 2024; Fu et al., 2023), yet research focusing on plasma apoEVs that are closely associated with aging remains scarce and unreported.

Lipids are critical structural and functional architects of cellular homeostasis. Recently, emerging evidence has defined novel roles of lipids in modulating organismal aging. Measuring the abundance of biological molecules in tissues and blood has been the corner stone of biomedical research and clinical diagnoses for decades, with blood-based assays offering convenience and minimal invasiveness (Ubaida-Mohien et al., 2023). Lipidomic analyses have been strongly associated with prevalent chronic conditions as well as predictive of incident events, including diabetes (Lauber et al., 2022), Alzheimer’s disease (Xu et al., 2020), cardiovascular disease (Seah et al., 2020), and cancer. Moreover, liquid biopsy, via noninvasive or minimally invasive procedures, provides a means to obtain real-time disease information. Advances in lipid analysis now enable comprehensive quantitative profiling and in-depth characterization from such samples (Shevchen et al., 2010). Accordingly, lipidomics has been applied to identify diagnostic lipid biomarkers in liquid biopsies and biofluid-derived extracellular vesicles (EVs) (Sagini et al., 2023).

Plasma lipidomics can effectively distinguish cancer patients from healthy individuals, as well as differentiate between benign and malignant conditions. Specific glycerolipid and sphingolipid species in plasma-derived EVs have been proposed as diagnostic biomarkers for colorectal cancer (Elmallah et al., 2022), and a distinct EV lipid signature can differentiate patients with hepatocellular carcinoma from those with cirrhosis alone (Sanchez et al., 2021). These findings illustrate how disease-induced alterations in the molecular cargo of plasma-derived EVs make them a promising source of biomarkers for various pathological conditions (Zhang et al., 2023). Additionally, age-related alterations in the circadian rhythms of the plasma lipidome have also been documented, including reduced rhythm amplitude, earlier acrophase, and disrupted synchronization between central and lipid metabolic cycles (Rahman et al., 2023).

Apoptotic extracellular vesicles (apoEVs) formed by membrane blebbing or protrusion with specific intracellular content distribution from apoptotic cells (Atkin-Smith et al., 2015), have emerged as active regulators of multiple biological processes, rather than merely cellular debris. Previous studies have reported that apoEVs can modulate macrophage and T-cell functions and promote tissue repair processes, including vascular protection and skin regeneration (Laing et al., 2018; Kou et al., 2018; Zernecke et al., 2009; Liu et al., 2020). Although ApoEVs hold promise as tools for the non-invasive diagnosis of age-related diseases, the differences in lipid profiles between plasma from young and aged individuals remain uncharacterized. Furthermore, it remains unclear which specific lipids play critical roles in influencing healthy longevity and the progression of age-related diseases. In this work, we hypothesize that aging triggers systemic lipid reprogramming within plasma ApoEVs. We aim to reveal these changes by comparing lipid profiles of plasma ApoEVs between young and aged people.

## 2 Materials and methods

### 2.1 Plasma collection

Whole-blood venous samples were collected from 50 healthy participants recruited from a routine health screening program. Of these, 24 individuals were confirmed to be free of major systemic diseases and thus eligible for inclusion in the study. Blood samples were collected into tubes containing EDTA as an anticoagulant with all procedures completed within 2 h of collection.

The study comprised 12 healthy aged participants (over 65 years old) and 12 healthy young participants (20–30 years old), with 6 males and 6 females in each group. Sample collection was performed at the Health Management Center of West China School of Public Health and West China Fourth Hospital, Sichuan University. The study protocol was approved by the medical ethics Committee of West China Fourth Hospital, Sichuan University (Approval No.: HXSY-EC-2022039).

### 2.2 ApoEVs extraction

ApoEVs were isolated from plasma supernatants of aged or young participants using a differential centrifugation protocol adapted from established methods (Crescitelli et al., 2013; Dieker et al., 2015; Phan et al., 2018), which has been validated in our previous study (Li et al., 2022). Briefly, equal-volume whole blood from young and aged subjects was used for ApoEV isolation to eliminate biases caused by inconsistent initial blood input. Plasma samples were centrifuged at 800 × g for 10 min at 4 °C. The supernatant was collected and further centrifuged at 3000× g for 20 min at 4 °C. The resulting precipitate was resuspended in PBS and centrifuged at 16,000 × g for 30 min at 4 °C. The pellet was resuspended in PBS and centrifuged again under identical conditions (16,000 × g, 30 min, 4 °C). The final pellet contained ApoEVs.

### 2.3 Characterization of ApoEVs

For nanoparticle tracking analysis (NTA), ApoEVs from young and aged plasma were serially diluted in particle-free PBS. Particle concentration as well as number-based and volume-weighted size-distribution profiles were acquired by NTA measurement.

For transmission electron microscopy (TEM), ApoEV suspensions were applied to carbon-coated copper grids, negatively stained with uranyl acetate, and air-dried. Ultrastructural features of ApoEVs were visualized under TEM, and representative images were collected.

For western blotting, total cell lysates and ApoEV samples were lysed with lysis buffer containing protease inhibitors. Protein quantification was performed, and equal protein amounts were subjected to SDS-PAGE electrophoresis and transferred to PVDF membranes. Membranes were blocked and probed with primary antibodies targeting cleaved Caspase-3 (380169,Zenbio), histone H3 (PA5-16183,invitrogen)and β-Actin (200068-8F10,Zenbio), then incubated with secondary antibodies. Target bands were detected via chemiluminescence.

### 2.4 Lipid analysis of ApoEVs

#### 2.4.1 Lipid extraction

Lipids were extracted using a MTBE method. Briefly, appropriate internal lipid standards were spiked into each sample prior to extraction. Samples were homogenized with 200 μL of water and 240 μL of methanol. Then, 800 μL MTBE was added, and the mixture was sonicated for 20 min at 4 °C, followed by incubation for 30 min at room temperature. After phase separation, the mixture was centrifuged at 14,000 × g for 15 min at 10 °C. The upper organic layer was carefully collected and evaporated to dryness under a gentle nitrogen steam.

#### 2.4.2 LC-MS/MS method for lipid analysis

Chromatographic separation was performed using reversed-phase liquid chromatography on a CSH C18 column (1.7 μm, 2.1 mm × 100 mm; Waters). The dried lipid extracts were reconstituted in 200 μL of 90% isopropanol/acetonitrile, centrifuged at 14,000 × g for 15 min, and a 3 μL aliquot of the supernatant was injected for analysis. The mobile phase consisted of solvent A (acetonitrile/water, 6:4, v/v) with 0.1% formic acid and 0.1 mM ammonium formate) and solvent B (acetonitrile/isopropanol, 1:9, v/v) with 0.1% formic acid and 0.1 mM ammonium formate). The gradient elution was programmed as follows: initial 40% B held for 3.5 min, increased linearly to 75% B over 9.5 min, then to 99% B over 6 min, and finally re-equilibrated at 40% B for 5 min. The flow rate was maintained at 300 μL/min.

Mass spectrometric analysis was carried out on a Q-Exactive Plus mass spectrometer operating in both positive and negative ionization modes. The electrospray ionization (ESI) source parameters were set as follows: source temperature, 300 °C; capillary temperature, 350 °C; spray voltage, 3.0 kV; and S-Lens RF level, 50%. The full scan range was set from m/z 200 to 1800.

#### 2.4.3 Lipid identification

Lipid identification was performed using the LipidSearch software, which contains a database of over 30 lipid classes and more than 1.5 million fragmentions. Mass tolerance for both precursorions and fragmentions were set to 5 ppm.

### 2.5 statistical analyses

Data were presented as mean ± standard deviation (SD). Differences between two groups were analyzed using an independent-samples t-test, and statistical differences among multiple groups were determined by one-way analysis of variance (ANOVA) using SPSS 22.0 software (SPSS Inc., Chicago, IL, United States). The principal component analysis (PCA) and partial least squares-discriminate analysis (PLS-DA) were performed using the online MetaboAnalyst5.0 platform. *P* values <0.05 were considered statistically significant.

## 3 Results

### 3.1 Phenotypic and molecular characterization of young- and aged-plasma-derived ApoEVs

We isolated apoptotic-derived extracellular vesicles (ApoEVs) from young and aged plasma and performed phenotypic and molecular characterization. Nanoparticle tracking analysis (NTA) demonstrated that both young and aged ApoEVs displayed size distribution profiles typical for apoptotic extracellular vesicles (Figure 1A). Transmission electron microscopy further confirmed the classic cup-shaped ultrastructure (Figure 1B). Collectively, these western blot data confirmed the apoptotic origin of our isolated ApoEVs supported by enriched cleaved Caspase-3. Histone H3 was abundant in total cell lysate, yet hardly detectable in either young- or aged-ApoEVs, indicating minimal loading of nuclear histone H3 cargo within these ApoEVs (Figure 1C).

**FIGURE 1:**
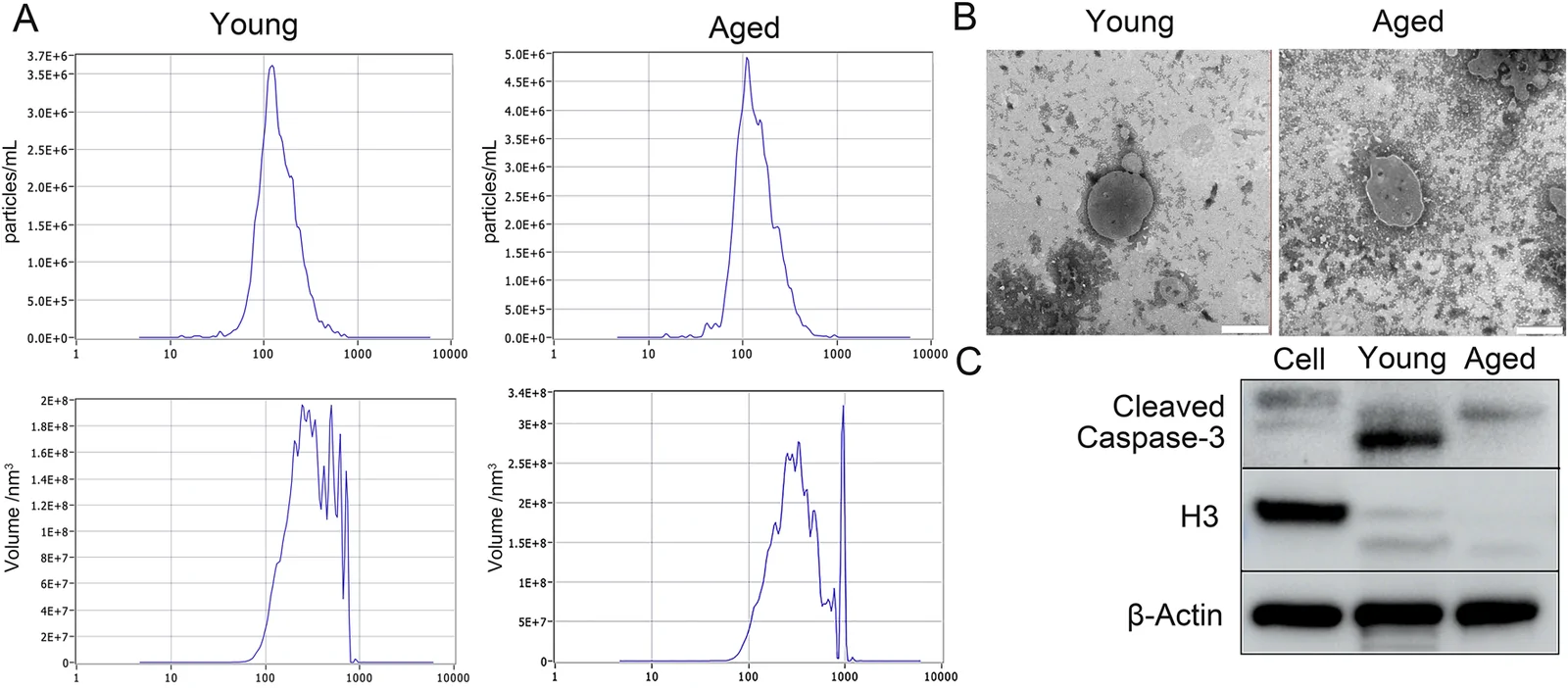
Identification of ApoEVs from young and aged cells. **(A)** NTA profiles showing particle number and volume-based size distribution of young- and aged-ApoEVs. **(B)** Representative TEM images of ApoEVs. Scale bar = 500 nm. **(C)** WB analysis for cleaved Caspase-3, histone H3(H3), and β-Actin in cell, young-ApoEVs and aged-ApoEVs.

### 3.2 Plasma ApoEVs Lipid composition

To investigate the lipid composition of plasma-derived ApoEVs from young and aged individuals, absolute quantitative lipidomic analysis was performed. In total, 953 individual lipid species, categorized into 32 distinct lipid classes, were identified, and quantitated in the lipidome. Phosphatidylcholine (PC) exhibited the highest number of lipid species at 187, followed by triglyceride (TG, 182) and phosphatidylethanolamine (PE, 123) (Figure 2A).

**FIGURE 2:**
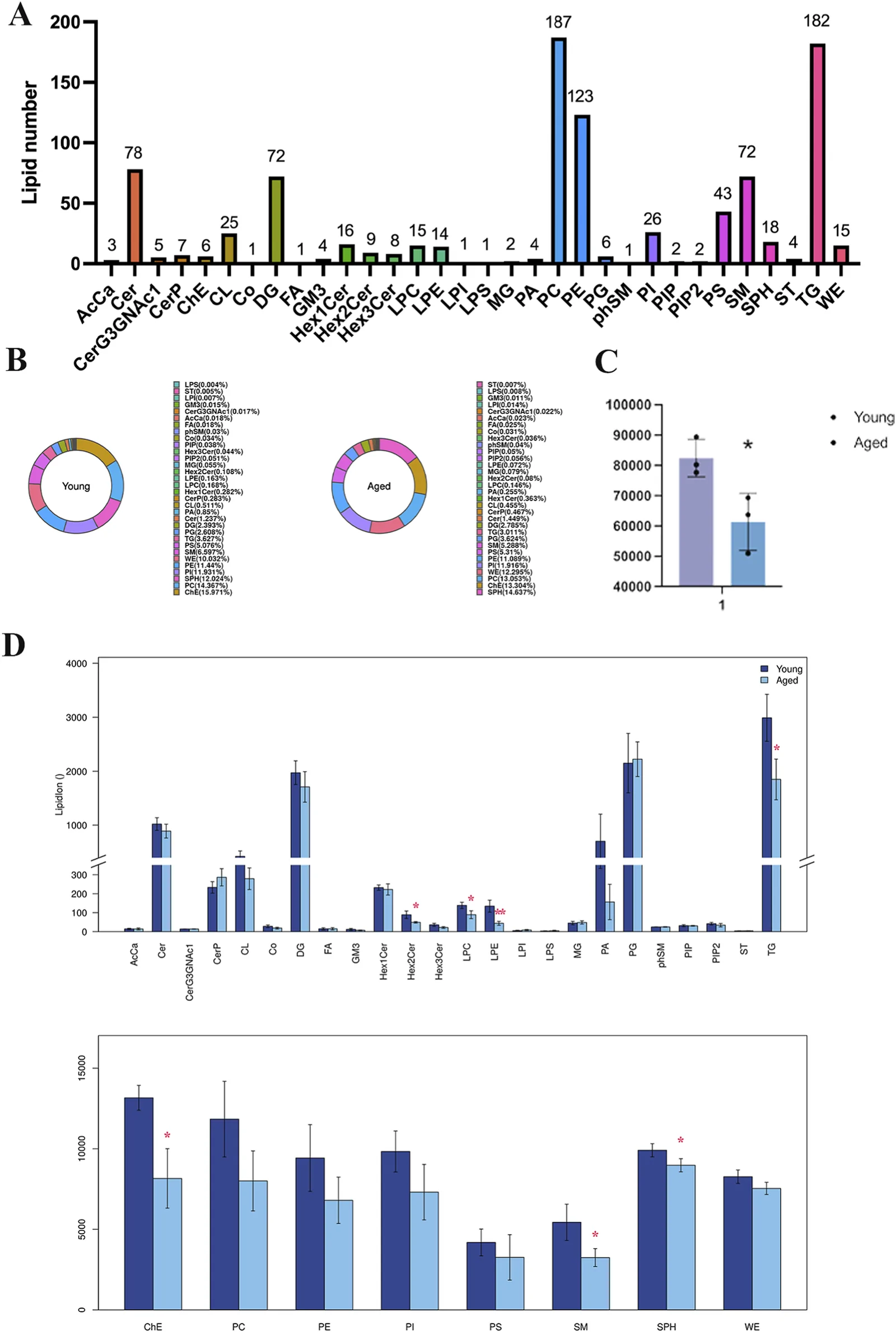
Classification and relative abundance of identified lipids. **(A)** Number of identified lipid class in young and aged groups. **(B)** Relative percentage of each lipid class in young and aged groups. **(C)** Total number of lipids in young and aged groups. **(D)** Comparison of lipid ion intensity across lipid classes in young and aged groups. Data are presented as mean ± standard deviation; *P < 0.05, **P < 0.01.

The relative percentages of lipid classes in ApoEVs from young and aged groups were calculated based on the absolute lipid quantities. Results showed that cholesterol ester (ChE), phosphatidylcholine (PC), sphingosine (SPH), wax esters (WE), phosphatidylinositol (PI) and phosphatidylethanolamine (PE) constituted the predominant lipid fraction in plasma ApoEVs, accounting for more than 70% of the total lipid (Figure 2B). Compared with the young group, the total number of lipid in the aged groups were decreased (Figure 2C). Hex2Cer, LPC, LPE, TG, Che, SM and SPH were significant different in young and aged groups Figure 2D.

### 3.3 Lipid profile of ApoEVs

To visualize group separation and identify differneces in lipid profile, principal component analysis (PCA) was first conducted on the plasma ApoEVs samples from young and aged groups. The three quality control (QC) samples clustered tightly in the PCA score plot (Figure 3A), indicating the good stability and reliability of the lipidomics platform. In addition, the analysis of PCA showed that the plasma samples from aged group were distinguished from those in the young group, indicating distinct lipid profiling patterns of plasm-dervied ApoEVs between young and aged groups.

**FIGURE 3:**
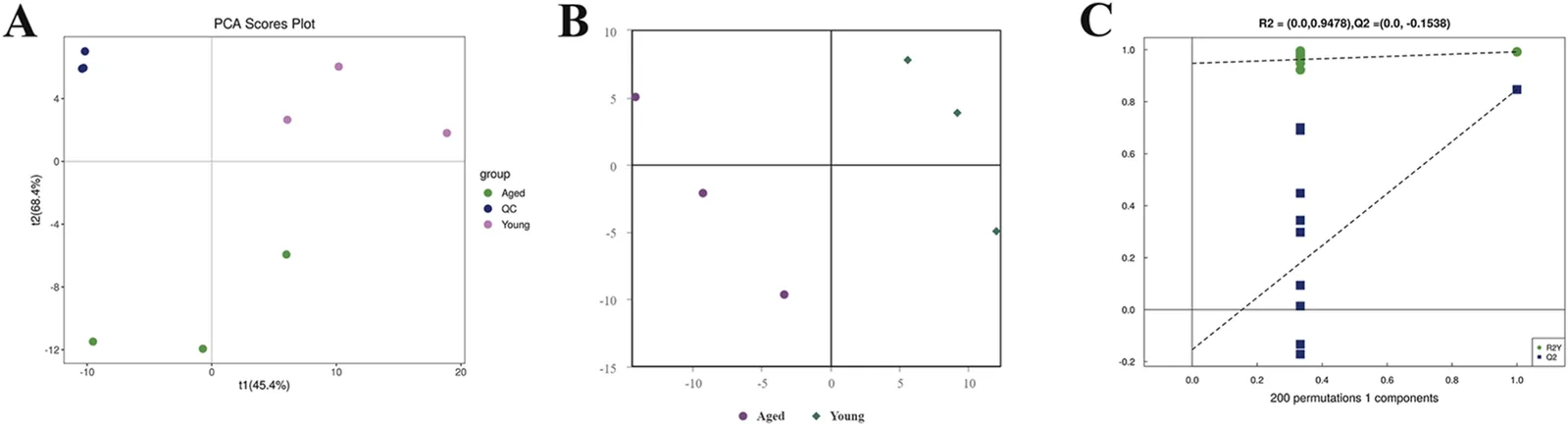
Lipidomic profiles between young and aged groups. **(A)** PCA scores plot showing the separation of lipidomic profiles between young and aged. **(B)** OPLS-DA scores plot displaying the discrimination between young and aged groups based on lipidomic data. **(C)** Permutation test validation of the OPLS-DA model with R^2^ and Q^2^ values, confirming the model’s validity.

To achieve enhanced separation and identify the lipids responsible for group discrimination, OPLS-DA (Orthogonal partial least squares-discriminant analysis),a supervised multivariate statistical method for group-separation analysis, was performed. The OPLS-DA model yielded the following parameters: R^2^X = 0.677, R^2^Y = 0.992 and Q^2^ = 0.847, indicating strong explanatory power (R^2^Y) and high predictive ability (Q^2^). A clear separation between the aged and young groups was observed in the OPLS-DA score plot (Figure 3B). To validate the model and exclude overfitting, a permutation test (200 times) was conducted, which confirmed the model’s robustness (Figure 3C). Together, the PCA and OPLS-DA results demonstrate a distinct difference in the lipid metabolic profile of plasma ApoEVs between the young and aged groups.

### 3.4 Difference of lipid species and pathway analysis

To determine the significantly changed lipid species in aged group, the lipids were screened based on VIP value >1 and *P* value (<0.05). In total, 27 lipid species with significant differences were screened out. Compared with the young group, the level of TG (15:0_16:0_18:2) was increased in the aged group, while the other 26 lipid species were significantly reduced (Figures 4A–D), and 18:1/18:2-containing lipids across TG, PC, ChE were significantly depleted in aged ApoEVs. The reduction was observed specifically in species containing oleic acid (18:1) and linoleic acid (18:2) acyl chains, indicating a degree of chain-length and unsaturation specificity in the age-associated lipid loss. This finding aligns with recent lipidomic evidence in the broader extracellular vesicle field. Patel and colleagues identified 247 exosome lipids in human plasma from young (20–26 years) and older (65–74 years) individuals, reporting that triglyceride species were downregulated in senescent cell-derived exosomes (Patel et al., 2025). Although that study focused on exosomes rather than ApoEVs, the parallel downregulation of TG species across different EV subtypes underscores a common aging-associated lipid signature. Furthermore, the coordinated depletion of structural (PC), storage (TG), and transport (ChE) lipids containing 18:1/18:2 in our ApoEV dataset extends these observations to the apoptotic vesicle compartment.

**FIGURE 4:**
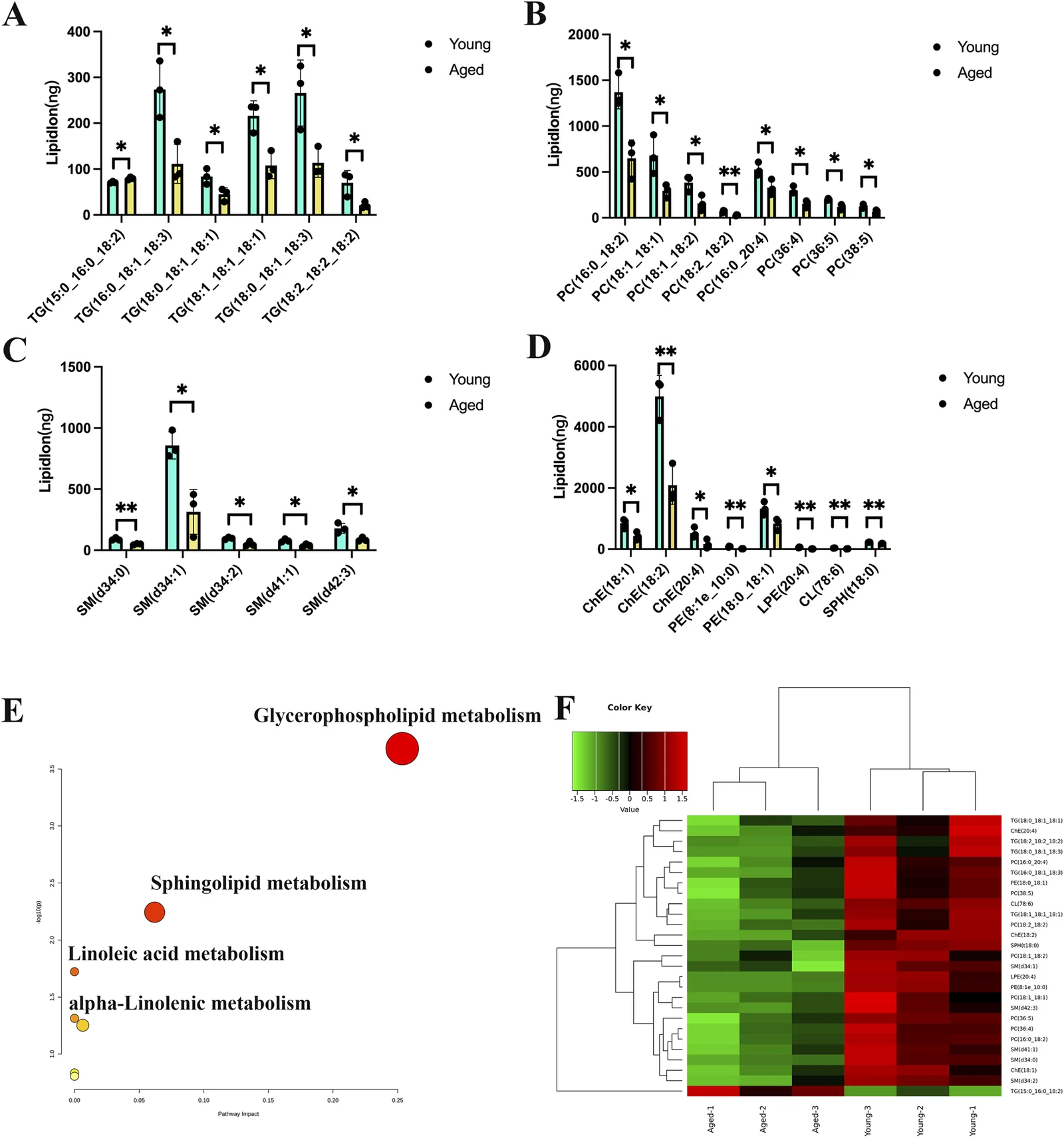
Identification of lipid species and pathway analysis. **(A–D)** Relative abundance of lipid species with significant differences between the young and aged groups, including TG **(A)**, PC **(B)**, SM **(C)**, and other lipids (ChE, PE, LPE, CL, SPH) **(D)**. **(E)** Pathway analysis. Glycerophospholipid metabolism, sphingolipid metabolism, linoleic acid metabolism, and alpha-linolenic acid metabolism are the top enriched pathways. **(F)** Heatmap showing the hierarchical clustering of differential lipid species between young and aged groups. Red indicates upregulation, green indicates downregulation, with the color key representing normalized abundance values; sample groups (aged, young) are clustered on the x-axis, and lipid species are clustered on the y-axis. Data are presented as mean ± standard deviation; *P < 0.05, **P < 0.01.

In addition, a heatmap with hierarchical clustering analysis of the 27 individual lipid species was plotted to effectively visualize the relationship and variations. It was found that the samples from the young group and aged group were gathered into two clusters in the dendrogram of the vertical axis in the heatmap (Figure 4F).

The selected lipid species were subjected to pathway analysis using MetaboAnalyst 6.0, which could provide clues to the affected biochemical pathways involving these lipids species. The results of the pathway analysis were shown in Figure 4E, where the bubble size and color represent the impact value of topology analysis and *p*-value of enrichment analysis, respectively. With the impact value above 0.10 and p-value less than 0.05, the glycerophospholipid metabolism bubble had the largest size and the deepest color.

### 3.5 Lipid chain length and saturation analysis

We analyzed the carbon chain length, and there was not significant difference between the young and aged groups, with the relative abundance of different chain-length species remained largely unchanged (Figure 5).

**FIGURE 5:**
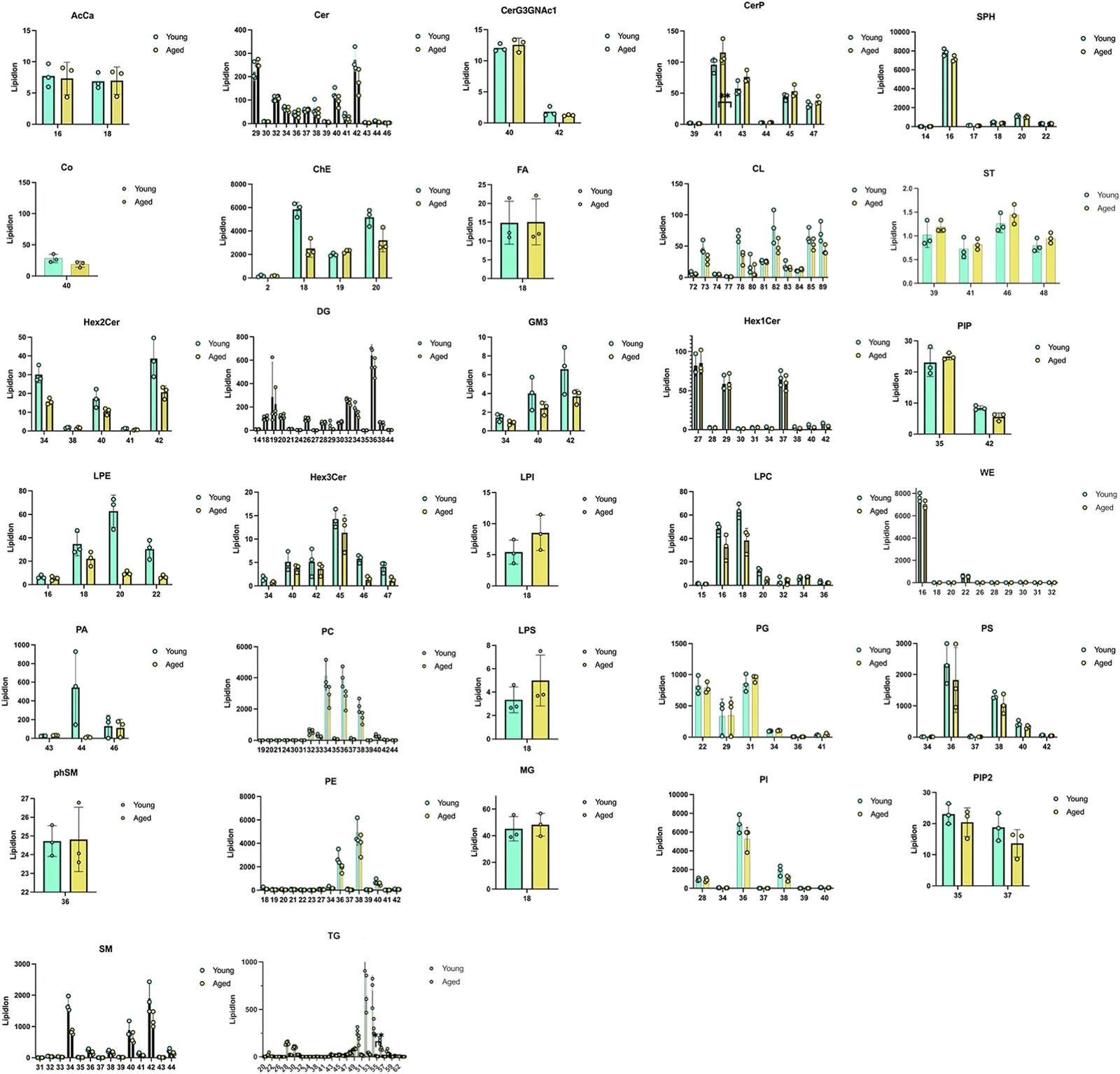
Alterations of lipid length in young and aged groups. Results of lipid length analysis. *P < 0.05, **P < 0.01.

Targeted lipidomic profiling revealed a significant depletion of lipid species across multiple classes in aged ApoEVs compared with the young group (Figures 6A–N). Compared with the young group, the aged ApoEVs exhibited significantly reduced levels of TG species containing 3, 4, 5 and 10 double bonds (Figure 6A), PC species containing 5 and 6 double bonds (Figure 6B), SM species containing 1 and 3 double bonds (Figure 6C), Cer species containing 2 and 3 double bonds (Figure 6D), ChE species containing 1 double bonds (Figure 6E), CL species containing 1, 5 and 6 double bonds (Figure 6F), Hex1Cer species containing 2 double bonds (Figure 6G), Hex3Cer species containing 3 and 4 double bonds (Figure 6H), LPC species containing 0,1,2 and 4 double bonds (Figure 6I), LPE species containing 2,3,4 and 5 double bonds (Figure 6J), WE species containing 0 double bonds (Figure 6K), PS species containing 6 and 7 double bonds (Figure 6L), SPH species containing 0 double bonds (Figure 6M), PIP species containing 10 double bonds (Figure 6N). The saturation of other lipids showed no obvious changes (Supplementary Figure S1).

**FIGURE 6:**
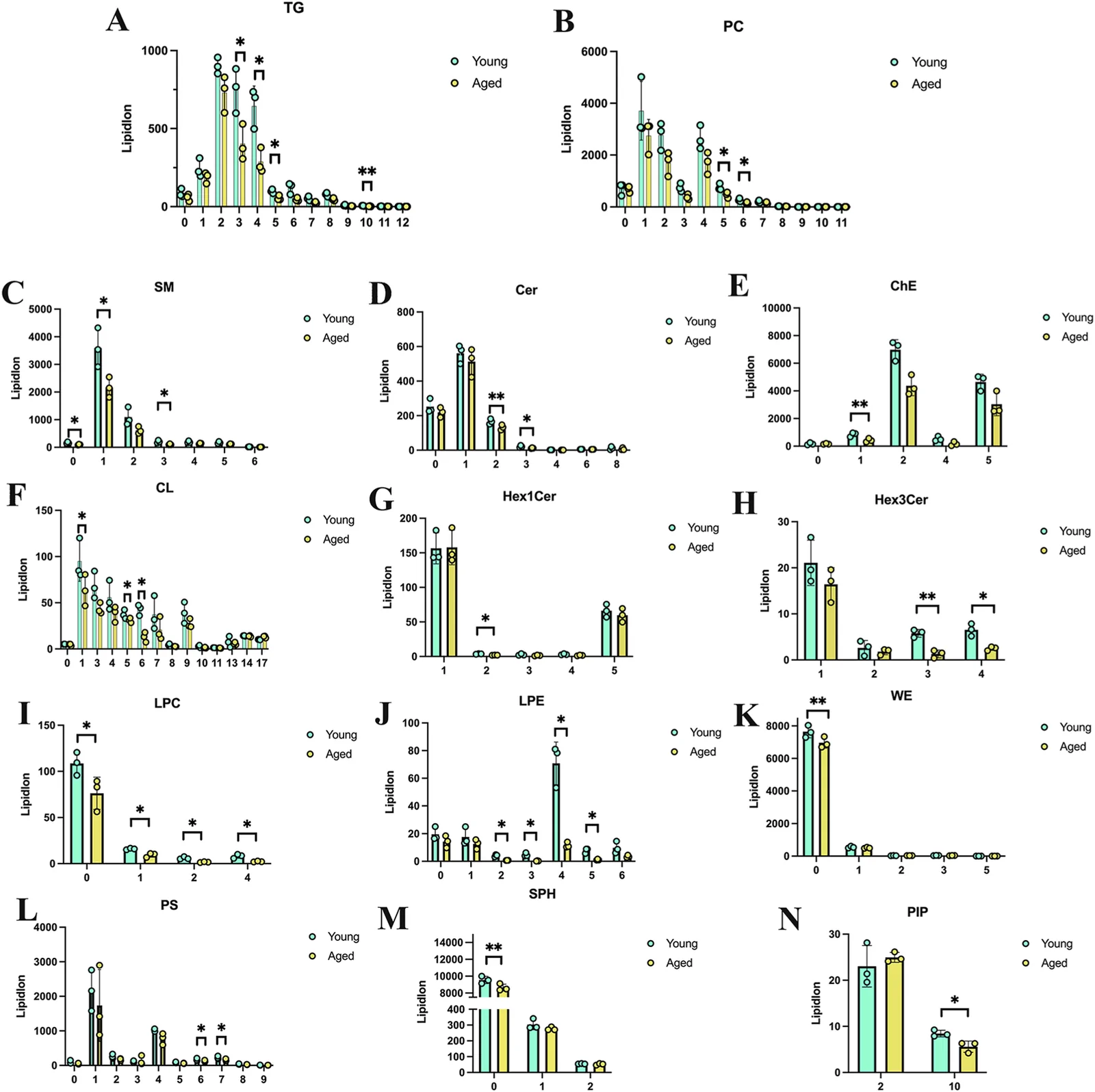
Alterations in lipid saturation between young and aged groups. Results of lipid chain saturation analysis. **(A)** Triglycerides (TG); **(B)** phosphatidylcholines (PC); **(C)** sphingomyelins (SM); **(D)** ceramides (Cer); **(E)** cholesteryl esters (ChE); **(F)** cardiolipins (CL); **(G)** monohexosylceramides (Hex1Cer); **(H)** trihexosylceramides (Hex3Cer); **(I)** lysophosphatidylcholines (LPC); **(J)** lysophosphatidylethanolamines (LPE); **(K)** wax esters (WE); **(L)** phosphatidylserines (PS); **(M)** phosphatidylethanolamines (PE); **(N)** phosphatidylinositol phosphates (PIP). *P < 0.05, **P < 0.01.

Although certain species with higher double-bond numbers (such as TG with 10 DB and PIP with 10 DB) were also reduced,the majority of the depleted species were those carrying low double-bond counts (ranging from 0 to 5), saturated or monounsaturated/polyunsaturated-with-low-unsaturation lipid entities. Consequently, the selective reduction of these saturated/monounsaturated lipid species led to a significantly decreased overall lipid saturation index within the aged ApoEV lipidome, indicating that the vesicular membrane and storage lipid compartments of aged ApoEVs shift toward a relatively more unsaturated state during the aging process.

## 4 Discussion

The shift of attention from disease prevention and treatment to the promotion of health and healthy aging carries an important implication for the direction in medical research and the future of medical practice. Numerous theoretical and empirical studies have sought to address how aging evolves at the mechanistic level. Nonetheless, what biological processes may cause aging at the mechanistic level remains a contentious, unresolved question. ApoEVs which are secreted from apoptotic cells harbor a spectrum of bioactive factors that play indispensable roles in systemic and tissue homeostasis (Fu et al., 2021). Alterations in ApoEVs can directly reflect the status of aging (Yang et al., 2025), as these vesicles mediate intercellular communication and participate in the clearance of apoptotic debris, a process progressively impaired with age (Xu et al., 2023). Characteristic alterations in lipid metabolism are now reported to correlate with age (Mutlu et al., 2021; Johnson and Stolzing, 2019; Zeng et al., 2024), and the changes in lipid profile can also accelerate the development of aging related phenotype, and serve as clinical indicators of underlying metabolic pathologies (Sharma and Diwan, 2023). Notably, lipidomic signatures have been increasingly recognized as reliable proxies for biological aging, as they integrate genetic, environmental, and physiological cues. Therefore, we investigated the lipidomic alterations of ApoEVs in plasma from young and aged individuals to uncover the mechanisms potentially underlying the initiation and progression of aging, and to identify early, intuitive indicators of age-related physiological changes.

The present lipidomic analysis of plasma apoptotic bodies during aging revealed a significant reduction in total lipid content, accompanied by consistent decreases in multiple key lipid subclasses, including Hex2Cer, LPC, LPE, TG, ChE, SM, and SPH. These alterations represent a characteristic lipid signature of aging and reflect profound disturbances in membrane structure, apoptotic signaling,and lipid metabolism. Sphingolipids, including SM, SPH, and Hex2Cer, are a vastly diverse class of lipids essential for proper cellular function regulation, particularly structure and signaling. There is strong evidence highlighting the role of sphingolipids in cellular senescence and aging (Li and Kim, 2021; Tang et al., 2022), but the precise mechanisms have yet to be elucidated. Sphingosine-1-phosphate (S1P), which promotes cellular proliferation and survival (Proia and Hla, 2015), is generated through the phosphorylation of SPH by SK1/2 (Adams et al., 2020). The downregulation of SK1/SK2 or decreased levels of cellular S1P have been shown to promote cellular senescence, consistent with our finding of reduced SPH levels in aged ApoEVs, which may impair S1P-mediated anti-senescence signaling (Cui et al., 2022).

Declines in LPC and LPE indicate dysregulated lysophospholipid metabolism in aging. Previous studies on lysophosphatidylcholines (LPCs) and aging have yielded inconsistent findings, which may be attributed to sex-specific differences, as reported in a recent systematic review showing LPC increases in aged women but decreases in aged men (Slade et al., 2021). Maekawa et al. observed elevated LPC metabolites in older women compared with younger counterparts (Maekawa et al., 2017). in contrast, other cohorts showed declining plasma or tissue LPCs with age (Beyene et al., 2020; Norris et al., 2015). Furthermore, decreased levels of TG and ChE reflect age-related disturbances in neutral lipid metabolism, lipid storage, and energy homeostasis.

Our lipidomic analysis revealed a distinctive remodeling pattern in circulating ApoEVs during aging: the carbon chain length of major lipid classes (TG, PC, SM, Che, PE, LPE, CL, and SPH) remained unaltered, while their overall fatty acid saturation significantly declined. This observation decouples two fundamental lipid structural properties—chain length and saturation—and highlights a selective, aging-driven perturbation in lipid desaturation dynamics within plasma ApoEVs.

The lipidomic landscape of human plasma across the lifespan has shown that glycerophospholipid metabolism is the most strongly correlated with metabolites (Liu et al., 2026). Our lipidomic profiling of plasma ApoEVs from aged and young individuals revealed that glycerophospholipid metabolism is the most profoundly dysregulated pathway in aging. This finding positions glycerophospholipid homeostasis as a critical node linking age-related physiological changes and aging.

The depletion of 18:1- and 18:2-containing species across TG, PC, and ChE in aged ApoEVs reveals a profound age-related remodeling of the plasma lipidome that extends beyond simple quantitative loss. This pattern is consistent with the lipidomic profiling by Patel et al. (2025), who demonstrated that senescence significantly downregulates multiple TG species in circulating exosomes and senescent cell-derived EVs.

Mechanistically, the depletion of 18:2-containing PC and ChE is particularly noteworthy. Linoleic acid (18:2) serves as the essential precursor for arachidonic acid and downstream oxylipins, while 18:1 (oleic acid) is crucial for maintaining membrane fluidity and buffering lipotoxicity via stearoyl-CoA desaturase 1 (SCD1). Their concurrent decline likely compromises the biophysical properties of the ApoEV membrane, thereby impairing efferocytosis, a process that is already defective in aging tissues (Poon and Ravichandran, 2024).

The age-dependent functional decline of ApoEVs has been directly demonstrated in regenerative contexts. Yan and colleagues showed that while both young and aged adipose stem cell-derived ApoEVs induced skin healing and reduced scar formation, young ApoEVs exhibited significantly higher proliferation, migration of fibroblasts and endothelial cells, and increased neo-angiogenesis ability compared to aged ApoEVs (Yan et al., 2024). We suggest that this functional decline may be partially underpinned by the lipid compositional alterations observed in our study, but direct causal evidence linking specific lipid species to functional outcomes remains to be established.

The age-associated decline in total lipids and fatty-acid saturation in plasma ApoEVs might reflect multifactorial metabolic reprogramming driven by mitochondrial dysfunction, impaired autophagy/lipophagy, peroxisomal failure, and dysregulation of PPARs/PGC-1α (Lopez-Otin et al., 2023). Although individual lipid changes may be compensatory (e.g., PLA2G2F-mediated lipid homeostasis), age-related “lipid drift”, cholesterol transport defects, lipid raft pathology, and ceramide accumulation, actively drives neurodegeneration and other aging pathologies. Based on previous studies, early adaptive changes are reversible,yet once past the critical tipping point of organelle dysfunction and chronic inflammation, systemic lipid decline becomes a maladaptive driver of aging. Our lipidomic signature (decreased Hex2Cer, LPC, LPE, TG, ChE, SM, SPH) likely reflects a post threshold state, positioning these lipids as both aging biomarkers and therapeutic targets (Yan et al., 2024).

Collectively, the comprehensive reduction in total lipids, multiple lipid subclasses and fatty acid saturation within plasma apoptotic bodies highlights systemic lipid reprogramming in aging. Decreased total levels of Hex2Cer, LPC, LPE, TG, ChE, SM, and SPH are features of aging. This signature not only deepens the understanding of aging-related lipid dysfunction and defective apoptotic clearance but also provides potential circulating lipid biomarkers for evaluating biological aging and age-related pathological processes.

## Data Availability

The raw data supporting the conclusions of this article will be made available by the authors, without undue reservation.
