# Supplementary material for: Quantitative lipidomics reveals Lipid perturbation of plasma apoptotic extracellular vesicles during physiological aging
Source: Front Cell Dev Biol. 2026 Sep 30;14:1853081. doi: 10.3389/fcell.2026.1853081 (PMC13661935; doi:10.3389/fcell.2026.1853081)
Supplement: Supplementary file 1 [file Supplementaryfile1.docx]

**Supplementary figures：**

**Figure S1.** Alterations of lipid saturation between aged and young groups. *P < 0.05, **P < 0.01.
